# Evaluation of Acne Quality of Life and Clinical Severity in Acne Female Adults

**DOI:** 10.1155/2010/410809

**Published:** 2010-07-27

**Authors:** Amal Kokandi

**Affiliations:** Rabigh College of Medicine, King AbdulAziz University, P.O. Box 42797, Jeddah 21551, Saudi Arabia

## Abstract

Acne is a common disease especially among teenagers. It has a considerable psychological impact on affected individuals. The aim of this paper was to assess if the effect of acne on acne-related quality of life is correlated to acne clinical severity. 112 university female students attending the university medical clinics with acne complaints were examined. Cardiff Acne Disability Index (CADI) was used to assess acne-related quality of life, and global acne grading system (GAGS) was used to assess clinical severity of acne. There was no correlation between acne severity (GAGS scoring system) and quality of life impairment as assessed by CADI score (*r* = 0.145, *P* = .127). Additionally, CADI score did not correlate with disease duration or age of patients. We therefore conclude that acne clinical severity alone does not affect acne-related quality of life changes. Many other factors might play a role.

## 1. Introduction

Acne vulgaris is a common disease with prevalence reaching up to 80% during adolescence [1]. The psychological effect of acne on patients can be considerable. The interaction of acne and psychosocial issues is complex and, in adolescence, can be associated with developmental issues of body image, socialization and sexuality. Previous studies on the psychosocial impact of acne have documented dissatisfaction with appearance, embarrassment, self-consciousness, and lack of self-confidence in acne patients. Social dysfunction has also been observed, including concerns about social interactions with the opposite gender, appearances in public, interaction with strangers, and reduced employment opportunities [2–4]. Furthermore, acne is associated with anxiety, depression [5], feel of anger [6], and lower body satisfaction [7]. It can be negatively associated with intention to participate in sports and exercise [8].

Additionally, mental health scores were reported to be worse for asthma, epilepsy, diabetes, back pain, arthritis, and coronary artery disease [9]. Psychological impact affects female patients more than male patients [5, 10]. Even suicidal ideation was found to be around 6-7% in acne patients [11, 12].

In this paper the acne-related disability was studied in relation to acne severity in female university students suffering from acne.

## 2. The Study

### 2.1. Subjects

Female students attending King Abdulaziz university medical administration dermatology clinic (female section) with acne complaints.

### 2.2. Methods

A medical history was taken in addition to the acne-related complaint. An assessment of acne was made using the global acne grading system or GAGS [13]. GAGS considers six locations on the face and chest/upper back, with a factor for each location based roughly on surface area, distribution, and density of pilosebaceous units. The severity was graded as mild if the score was 1–18, moderate with scores from 19 to 30, severe with scores from 31 to 38, and as very severe if the score is more than 38.

The Cardiff Acne Disability Index (CADI) was completed by the physician [14]. CADI is a short, 5-item questionnaire derived from the longer Acne Disability Index [15]. It is usually completed in one minute [16]. The total score can range from 0 to 15.

### 2.3. Statistics

The statistical analysis was performed using the SPSS software version 10.0. Spearman Rank Correlation test was used for correlation between the variables.

## 3. Results and Discussion

### 3.1. Results

One hundred and twelve cases were examined. Age ranged from 18 to 40 years (median 24 years). Disease duration ranged from 1 month to 20 years (median 5 years). Seventeen (15% of the total cases) did not use any treatment for acne previously. Roaccutane was used previously by 8 (7% of the total cases). CADI score ranged from 1 to 15. GAGS ranged from 8 to 37. Most cases (82) were classified as mild acne (73.2% of cases), 28 were classified as moderate severity (25% of cases), 2 were classified as severe acne (1.8% of cases), and no cases were classified as very severe (see Table 1).

**Figure 1:**
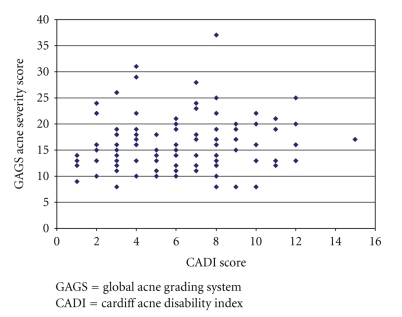
Distribution of cases according to GAGS acne severity score and CADI disability index score.

There was no correlation between CADI score and GAGS acne severity score (*r* = 0.145, *P* = .127). There was no correlation between the CADI score and age of patients (*P* = .869, *r* = −0.016), and no correlation was found between the CADI score disease duration as well (*P* = .941, *r* = −0.007). Figure 1 illustrates the distribution of cases according to GAGS acne severity score and CADI disability index score.

### 3.2. Discussion

Acne has a considerable psychological impact on affected individuals [17]. Previous studies have shown that acne is associated with significant morbidity and decrease in health-related quality of life. Both general practitioners and dermatologists were reported as having poor comprehension of the psychological implications of skin diseases, being insensitive to their patients' emotional suffering, and trivialising participants' disease [18]. In this study the degree of acne-related quality of life changes on patients were studied in relation to acne severity. The global acne grading system (GAGS) was used to assess acne severity and the Cardiff Acne Disability Index (CADI) was used to assess the impact of acne on quality of life.

Different methods exist for assessing acne severity. At least 25 methods were described [19]. GAGS was used because it is easy to use and does not require special training. Considering quality of life scales, CADI is the easiest scale to use in routine dermatology practice [20]. Most cases in this study were classified as mild or moderate severity. This is because the sitting is not a referral centre. 

CADI was found not to correlate with acne severity as assessed by GAGS. A recent study showed similar result in Hong Kong, which found no correlation between GAGS acne severity score and CADI score [21]. A similar result was obtained using different scoring system for acne severity, ECLA scale (Echelle de Cotation des Lésions d'Acné or Acne Lesion Score Scale). As ECLA overall scale did not correlate with CADI score in French population sample [22], similarly in Turkish sample of patients there were no significant relationships between acne severity and AQOLS/DLQI [23]. Additionally, anxiety or depression (assessed by HAD hospital anxiety and depression scale) were reported not to correlate with acne severity [24]. On the other hand, Walker and Lewis-Jones [25] found that a good correlation was found between CADI and other quality of life measures (CLDQI: Children's Dermatology Life Quality Index) in Scottish teenagers [25].

In this paper, age and disease duration did not correlate with quality of life as well. Consequently, it has been found in our study that the quality of life in acne patients can be affected by reasons other than acne severity. The reasons can be social, emotional, personality type, presence of scarring and school-, or job-related problems.

We therefore conclude that acne clinical severity alone does not affect quality of life. Many other factors might play a role. Each patient should be treated individually taking into consideration that mild disease does not mean little effect on quality of life.

## Figures and Tables

**Table 1:** GAGS (global acne grading system) score distribution.

Grade of acne severity	GAGS score	Number of cases
Mild	1–18	82
Moderate	19–30	28
Severe	31–38	2
Very severe	>38	0

Total		112
